# Attenuation of A(H7N9) influenza virus infection in mice exposed to cigarette smoke

**DOI:** 10.1038/s44298-024-00026-4

**Published:** 2024-03-25

**Authors:** Satoshi Fukuyama, Jason E. Shoemaker, Dongming Zhao, Noriko Nagajima, Yuriko Tomita, Tadashi Maemura, Tiago Jose da Silva Lopes, Tokiko Watanabe, Seiya Yamayoshi, Hideki Hasegawa, Yoshihiro Kawaoka

**Affiliations:** 1 Division of Virology, Institute of Medical Science, University of Tokyo, Shirokanedai, Minato-ku, Tokyo, 108-8639 Japan; 2 Department of Chemical and Petroleum Engineering, Swanson School of Engineering, University of Pittsburgh, Pittsburgh, PA USA; 3 Department of Computational & Systems Biology, University of Pittsburgh, Pittsburgh, PA USA; 4 Department of Pathology, National Institute of Infectious Diseases, Tokyo, Japan; 5 Influenza Virus Research Center, National Institute of Infectious Diseases, Tokyo, Japan; 6 Department of Molecular Virology, Research Institute for Microbial Diseases, Osaka University, Osaka, Japan; 7 Center for Advanced Modalities and DDS, Osaka University, Osaka, Japan; 8 Center for Infectious Disease Education and Research, Osaka University, Osaka, Japan; 9 Department of Pathobiological Sciences, School of Veterinary Medicine, University of Wisconsin–Madison, Madison, WI USA; 10 International Research Center for Infectious Diseases, Institute of Medical Science, University of Tokyo, Tokyo, Japan; 11 The Research Center for Global Viral Diseases, National Center for Global Health and Medicine Research Institute, Tokyo, Japan; 12 The University of Tokyo, Pandemic Preparedness, Infection, and Advanced Research Center, Tokyo, Japan

**Keywords:** Virology, Immunopathogenesis

## Abstract

Influenza A(H7N9) virus showed high pathogenicity in humans when it emerged in 2013. Cigarette smoke (CS) causes pulmonary diseases including bronchitis, emphysema, and lung cancer. Although habitual smoking is thought to increase the risk of severe seasonal influenza virus infection, its effect on A(H7N9) virus infection is poorly understood. Here, we employed a mouse model of long-term exposure to CS to investigate the effect of CS on the pathogenicity of A(H7N9) virus infection. Unexpectedly, body weight loss for mice exposed to CS was milder than that for mock-treated mice upon A(H7N9) virus infection. CS exposure improved the survival rate of A(H7N9) virus-infected mice even though virus titers and pathological changes in the lungs were not significantly different between CS-exposed and control mice. Microarray analysis showed that CS-exposure activates cytokine/chemokine activity, immune response, and cell cycle activities that resemble reactivities against A(H7N9) virus infection. Therefore, under conditions where cytokine and chemokine expression in the lungs is already high due to CS exposure, the enhanced expression of cytokines and chemokines caused by A(H7N9) virus infection might be less harmful to the organs compared to the rapid increase in cytokine and chemokine expression in the air-exposed mice due to the infection. CS may thus induce immunoregulatory effects that attenuate severe pulmonary disease during A(H7N9) virus infection. However, these findings do not support CS exposure due to its many other proven negative health effects.

## Introduction

The first human case of infection with avian-origin influenza A(H7N9) virus was reported in China in February 2013^1^. Since then, five epidemic waves of A(H7N9) infection, comprising 1568 confirmed human cases and 615 deaths, have occurred in eastern China^2^. The A(H7N9) virus is a reassortant of three avian viruses^3^. Studies of primary isolates from humans with A(H7N9) infection have revealed that the hemagglutinin (HA) of A(H7N9) viruses binds to human-type receptors (α2,6-linked sialosides), conferring infectivity to human respiratory organs^4,5^. During the fifth wave, from 2016 to 2017, highly pathogenic A(H7N9) virus emerged in poultry and infected humans^6,7^. This highly pathogenic A(H7N9) virus had increased pathogenicity in mammals such as mice and ferrets compared with the earlier A(H7N9) viruses and could transmit among ferrets by respiratory droplets^8^. To combat this threat, China undertook a national vaccination strategy with the H5/H7 bivalent avian influenza vaccine. It effectively controlled outbreaks and circulation of A(H7N9) viruses in poultry, significantly reduced the virus load in the environment, and prevented further A(H7N9) virus infections in humans. Since October 2017, only four human cases have been reported, with the most recent case being reported in March of 2019^9^. Although human cases have clearly decreased, careful monitoring of A(H7N9) virus is essential to ensure a prompt response to the emergence of an isolate with pandemic potential.

Epidemiologic studies have shown that many patients with confirmed A(H7N9) virus infection were exposed to poultry in the live poultry markets and backyards^10,11^. Studies by Cowling et al. and Zhou et al. in different seasons showed that people infected with A(H7N9) virus tended to be older than people infected with other avian influenza viruses such as the A(H5N1) virus [i.e., median age of 57 years for A(H7N9) cf. median age of 26 years for A(H5N1)]^12,13^. These epidemiological features of A(H7N9) virus were common among all five epidemic waves^14^. In addition to age and poultry exposure, susceptibility to and mortality following A(H7N9) virus infection are influenced by underlying medical conditions including chronic obstructive pulmonary diseases (COPD), cardiovascular diseases, diabetes, and immunosuppression^11^. The effect of cigarette smoke (CS) on A(H7N9) virus infection in humans is unclear because the subpopulation of smokers among the cases of A(H7N9) virus infection was too low to analyze epidemiologically compared with controls^11^.

CS exposure in humans is associated with an increased risk of seasonal influenza virus infection and severe symptoms^15–17^. A meta-analysis showed that current smokers were more than 5 times likely to develop influenza than nonsmokers^18^. CS triggers the release of pro-inflammatory cytokines (e.g., IL-8 and TNF-α) by resident immune cells and epithelial cells, resulting in elevated neutrophil and macrophage levels in the lungs^19–22^. In CS-exposed mouse model studies, seasonal influenza virus infection resulted in worse disease outcomes compared to non-smoking controls: CS-exposed mice showed severe weight loss^23–26^ and higher mortality^22,24,26,27^ compared to non-CS infected mice. The poorer outcomes in the CS mouse model could be due to suppression of antiviral immune responses by CS exposure that allow superior propagation of the virus and/or that the viral infection triggers an exaggerated inflammatory response^28^. Yet, Han et al. report that CS exposure suppresses the inflammatory response induced by seasonal influenza virus infection in a nicotine-dependent manner and reduces body weight loss and mortality^29^. These studies employed a sublethal dose of seasonal H1 or H3 influenza virus for a CS-exposure model and measured specific cytokine and chemokine responses with or without infection and CS exposure. Therefore, we analyzed the effect of CS exposure on the pathogenicity of A(H7N9) virus infection, which is more virulent than seasonal H1 and H3 influenza virus infection. Furthermore, we performed a microarray analysis to draw a complete picture of the inflammatory response to A(H7N9) virus infection after CS exposure.

## Results

### CS exposure inhibits body weight loss upon A(H7N9) virus infection

**Fig. 1:**
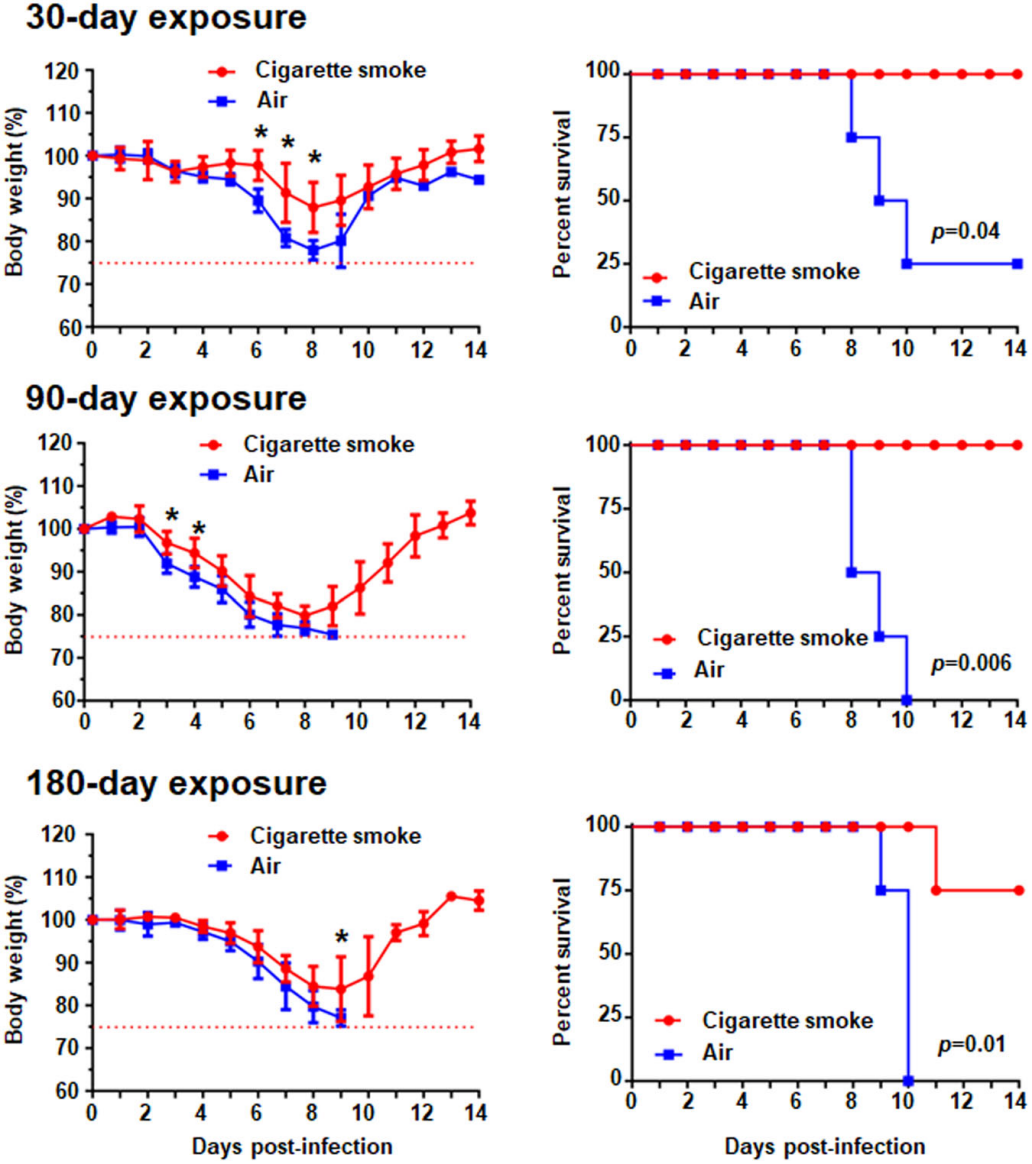
Body weight changes and survival rates of CS- or air-exposed mice after A(H7N9) virus infection. Mice (*n* = 4 per group) that were exposed to CS or air for 30, 90, or 180 days were infected with 10^3^ PFU of A(H7N9) virus. Average body weight with SD (*p < 0.05) and survival monitored daily for 14 days were shown.

To examine whether CS affects the pathogenesis of A(H7N9) virus infection, we used a CS-exposure mouse model, which has been established previously^30,31^. Air-exposed mice and CS-exposed mice were infected with A(H7N9) virus and their body weight changes were monitored for 14 dpi. Air-exposed mice showed severe body weight loss and died within 10 dpi (Fig. 1). In contrast, 100%, 100%, and 75% of mice exposed to CS for 30 days, 90 days, and 180 days, respectively, survived with slightly less body weight loss. These results suggest that CS exposure for 30, 90, and 180 days consistently reduces the pathogenicity of A(H7N9) virus in mice.

**Fig. 2:**
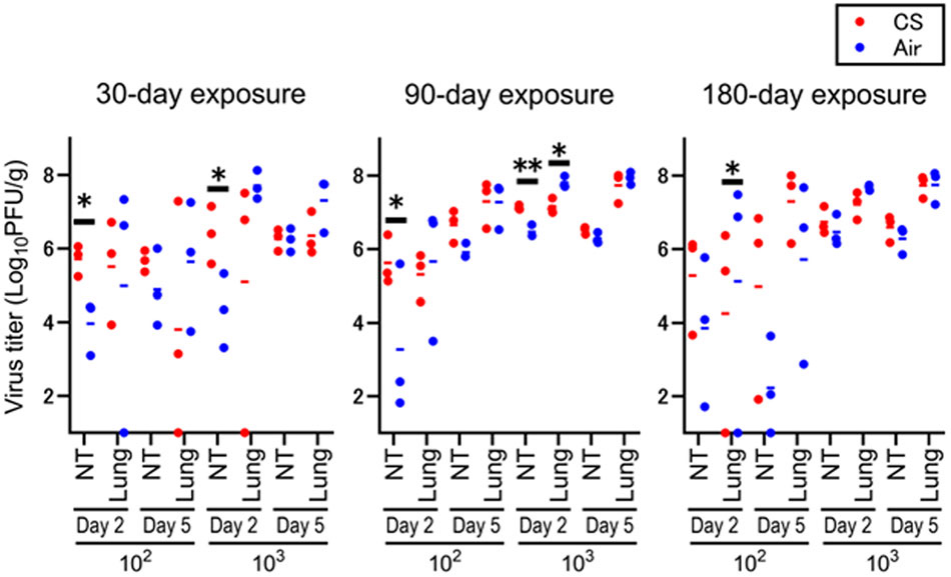
Virus titers in the lungs of CS- or air-exposed mice after A(H7N9) virus infection. Mice (*n* = 3 per group) were exposed to CS or air for 30, 90, or 180 days. Nasal turbinates and lungs were isolated from mice at 2 or 5 dpi with 10^2^ or 10^3^ PFU of A(H7N9) virus. Virus titers with average bars in these tissues were determined by use of plaque assays with MDCK cells. (*p < 0.05 and **p < 0.01)

We next measured virus titers in the nasal turbinates and lungs of the CS- or air-exposed mice at 2 and 5 dpi with A(H7N9). Virus titers in nasal turbinates at 2 dpi with A(H7N9) virus were significantly higher in CS-exposed mice than those in air-exposed mice (Fig. 2). In contrast, virus titers in lung tissues did not differ between CS- and air-exposed mice upon A(H7N9) virus infection except that the virus titer on 2 dpi with 10^3^ PFU of virus in the mice exposed to CS for 90 days was significantly lower than that of the air-exposed mice. These data indicate that CS exposure might enhance A(H7N9) virus replication in the nasal turbinates especially at 2 dpi.

### CS exposure attenuates lung inflammation in A(H7N9) virus-infected mice

**Fig. 3:**
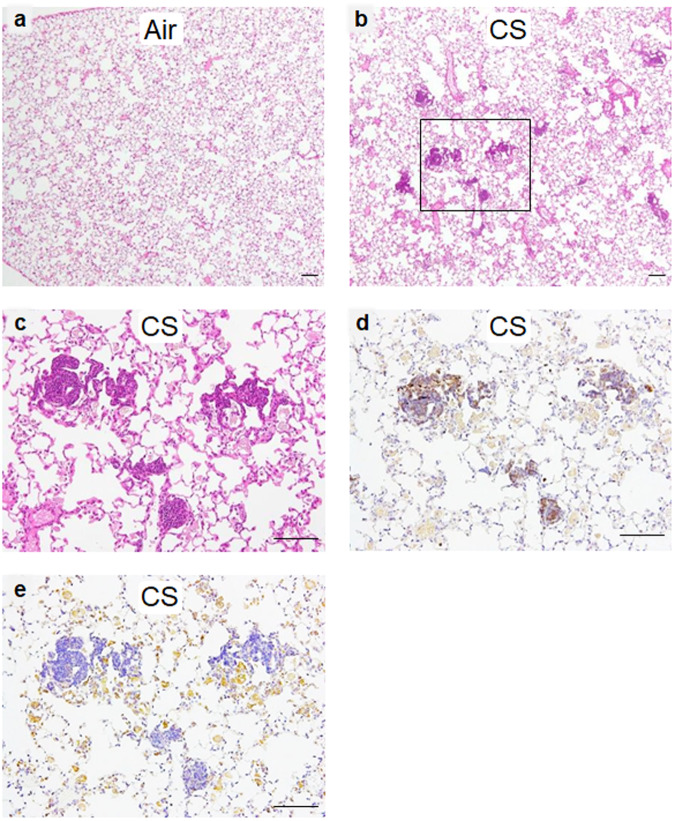
Histological characteristics of the lungs of mock-infected mice exposed to air or CS. Lungs were isolated from mice exposed to CS or air for 180 days. H&E staining of fixed lung tissues from air-exposed mice (**a**), and CS-exposed mice (**b**). **c** Enlarged image of the rectangle in **b**. Immunohistochemistry of serial sections of CS-exposed mice was assessed by staining the sections with a mouse anti-B220 antibody (**d**) and a mouse anti-Mac-3 antibody (**e**). B220 and Mac-3 are markers for pan-B cells and macrophages, respectively. Scale bars, 100 µm.

To address the pathological effect of A(H7N9) virus infection, we histologically analyzed lung tissues from mice exposed to CS or air after A(H7N9) virus infection. Since the observed reduced pathogenicity of A(H7N9) virus was similar under all three exposure conditions (see Fig. 1), we focused on the 180 day-exposure condition for further analysis. In mock-infected mice, there were no significant differences in emphysema-like changes in the lungs of CS-exposed mice compared to the lungs of air-exposed mice. Several small lymphoid follicles along the bronchioles and alveolar ducts were detected in the CS-exposed mice (Fig. 3b), whereas such pathological changes were not apparent in the air-exposed mice (Fig. 3a). Immunohistochemistry using antibodies against B220 and Mac-3, which are markers for a pan-B cells and macrophages, respectively, showed that these follicles contained accumulated B220+ cells (Fig. 3d), and high numbers of Mac-3+ cells were observed throughout the alveoli (Fig. 3e). The follicles were likely induced by the CS-exposure; however, their pathological significance is unknown.

**Fig. 4:**
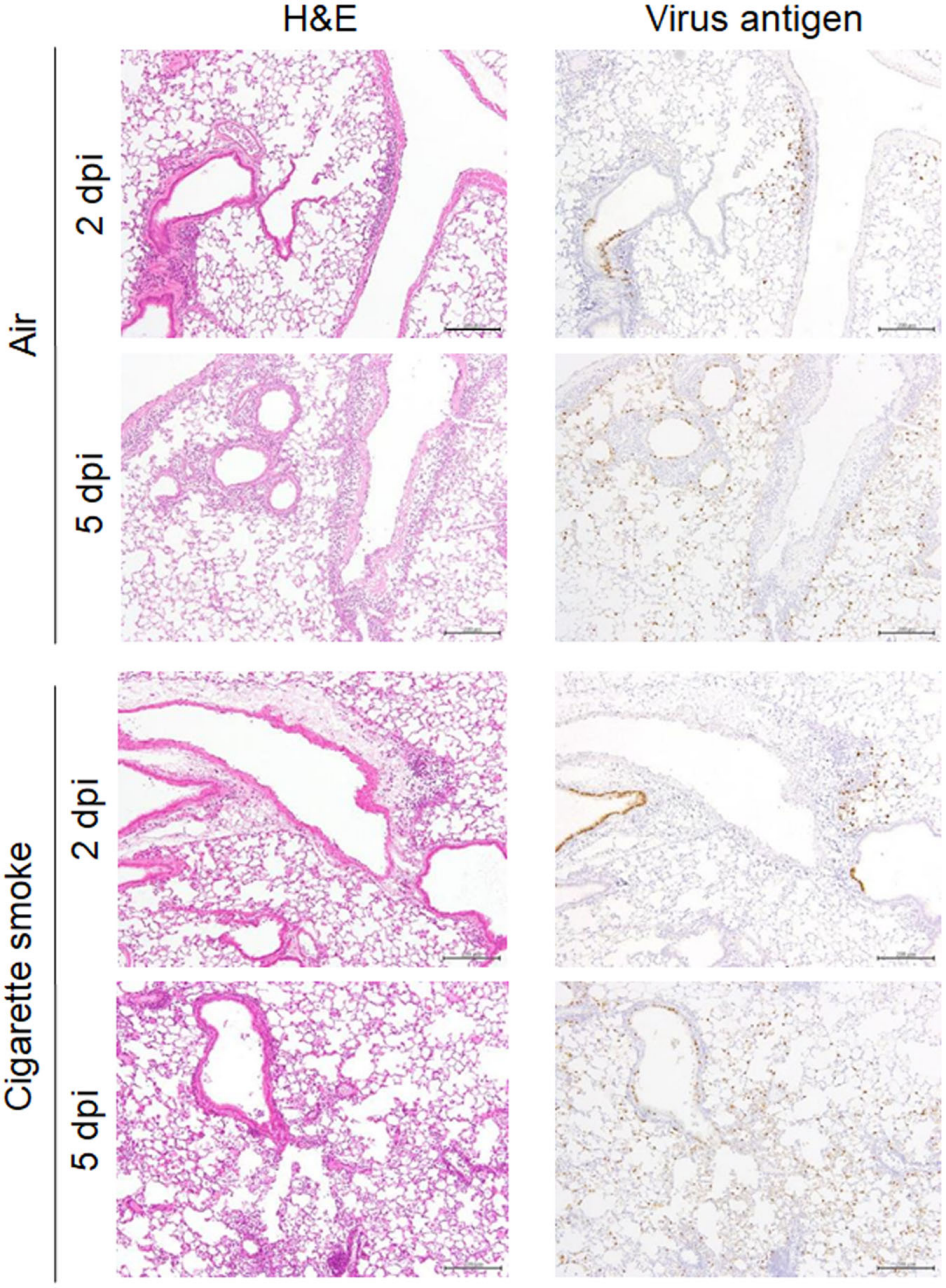
Lung pathology of CS- or air-exposed mice after A(H7N9) virus infection. Lung tissues of mice that were exposed to CS or air for 180 days were isolated at 2 or 5 dpi with 10^3^ PFU of A(H7N9) virus. H&E staining and immunohistochemistry using anti- type A influenza virus NP antibodies were performed on fixed lung tissues. Scale bars, 200 µm.

We next compared the pathology of the lungs between the air- and CS-exposed mice at 2 and 5 dpi with A(H7N9) virus. At 2 dpi, inflammatory cells accumulated along the bronchus and blood vessels at a similar level and the number of inflammatory cells in the alveoli region was similar between the CS-exposed mice and the air-exposed mice (Fig. 4). Virus antigen-positive cells were detected among the bronchial epithelial cells and type II pneumocytes of both the air- and CS-exposed mice on 2 dpi. On 5 dpi, the number of virus antigen-positive alveolar type II pneumocytes had increased in the lungs of both the air- and CS-exposed mice compared with 2 dpi. These pathological data indicate that the inflammatory response and virus distribution are similar in the lungs of CS-exposed and air-exposed mice after A(H7N9) virus infection. Overall, the histological changes caused by the CS exposure make this evaluation difficult.

### CS exposure alters the inflammatory response upon A(H7N9) virus infection

To analyze the influence of CS exposure on the host response, we performed a gene expression microarray of the lungs of air- and CS-exposed mice infected with 10^3^ PFU of A(H7N9) virus. The number of differentially expressed probes of the air-exposed mice at 5 dpi was increased from that at 2 dpi (Supplementary Fig. S1). There were considerably fewer differentially expressed probes in the lungs of CS-exposed mice both at 2 and 5 dpi compared with those of air-exposed mice, especially at 90 and 180 days of CS exposure (Supplementary Fig. S1). As our primary interest is how habitual smoking impact infection, we focused our statistical analysis on interaction effects. This analysis identifies genes that are differentially expressed due to infection depending on the CS-exposure status of the animal. We performed a two-way ANOVA to assess interaction effects between CS exposure and infection status on gene expression. This analysis revealed a statistically significant interaction (Itrx) between the effects of smoke exposure and infection status (FDR < 0.01 using the F-statistic to evaluate significance across different exposure periods) for 538 probes mapping to 461 unique gene entrez IDs, meaning that the expression of these 461 genes was affected by both CS exposure and infection.

**Fig. 5:**
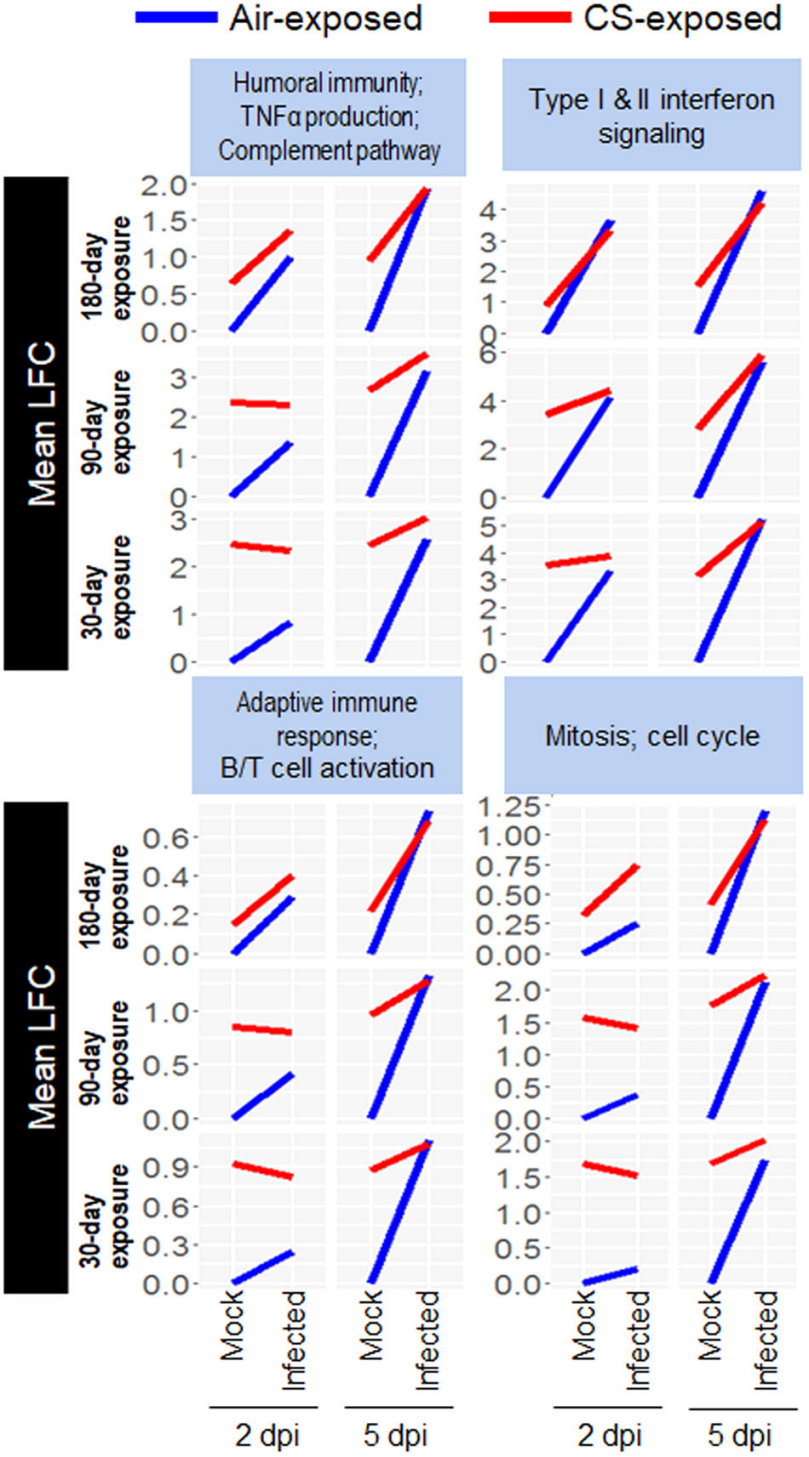
Clustered genes significantly impacted by smoking and infection status. Genes with a statistically significant interaction between the effects of smoke exposure and infection status (two-way ANOVA; FDR < 0.01) were clustered by using their gene expression across experimental conditions (*n* = 3 per condition). Shown is the mean log fold change (LFC) relative to mock-infected, air-exposed animals for all genes in each cluster. Gene ontology analysis was applied to the genes in each cluster to identify significant functional associations (a complete list of all enriched ontologies is available in Supplementary Table S1).

To characterize the differences between air-exposed and CS-exposed animals, the 461 differentially expressed genes where clustered horizontally and functional enrichment analysis was performed by using ToppCluster. Differences in gene expression across conditions were illustrated by using the mean gene expression of all genes assigned to each cluster, resulting in the identification of four clusters. Functional enrichment analysis revealed that these clusters contained genes associated with: (i) humoral immunity, TNF-α production, and complement pathway; (ii) type I & II interferon signaling; (iii) adaptive immune response and B/T cell activation; and (iv) mitosis and cell cycle (Supplementary Table S1 and Fig. 5). Analysis of cluster gene expression dynamics across conditions showed that there were significant differences between air-exposed and CS-exposed animals in the mock samples, but that the differentially expressed genes between the two cohorts became similar upon A(H7N9) virus infection, especially at 5 dpi (Fig. 5). These data indicate that CS exposure activates cytokine/chemokine activity, immune response, and cell cycle activities that resemble reactivities against A(H7N9) virus infection.

Finally, network-based analysis of the 461 differentially expressed genes was employed to identify key factors contributing to the expression profile in CS-exposed mice. This analysis suggested that several common immune regulators (e.g., TNFRSF1A, TNF-α, IL-1β, TNFSF12, EGR1, and CHUK) are differently regulated in CS-exposed mice (Supplementary Fig. S2). In particular, TNF-α was identified as a potential upstream regulator of the differentially expressed genes and was also observed to be primarily up-regulated in the lung by CS exposure (Supplementary Fig. S2). Several differentially expressed chemokines, including CXCL6, CCL9, CCL2, CCL3L1/CCL3L3, IL12b, SSA1, and MADCAM1, which affect phagocyte migration and lymphocyte movement, were regulated by TNF-α (Supplementary Fig. S3). Therefore, TNF-α may be a key regulator for protection against A(H7N9) virus infection in the CS-exposed mice.

## Discussion

Cigarette smoking is a widely known risk factor for numerous diseases such as lung cancer^32^, COPD^33^, and cardiovascular disease^34^. CS is also reported to increase the risk of respiratory infectious diseases caused by viruses such as influenza virus, human rhinovirus, and respiratory syncytial virus^15,17,35,36^. Contrary to epidemiological evidence, our study using a CS-exposed mouse model showed that CS exposure reduced the virulence of A(H7N9) virus without a significant reduction in virus titers. Highly pathogenic influenza viruses such as A(H7N9) and A(H5N1) viruses induce aberrant production of proinflammatory cytokines and chemokines including TNF-α, IL-6, and MCP-1 in the lungs of mice and cynomolgus macaques^4,37^, which leads to tissue damage through infiltration and activation of inflammatory cells^38^. However, our microarray analysis revealed that the expression of cytokines and chemokines was upregulated by CS exposure alone and the expression of cytokines and chemokines of air-exposed mice after A(H7N9) virus infection were similar to those of CS-exposed mice. These results indicate that when cytokine and chemokine expression in the lungs is already high due to CS exposure prior to infection, although their expression could be further enhanced by the viral infection, the increase rate of their expression levels in CS-exposed mice was modest compared to that in the control mice, suggesting that the rapid increase in cytokines and chemokines seen in air-exposed mice upon viral infection may be harmful to the respiratory organs, especially the lungs. Furthermore, since both CS exposure and A(H7N9) virus infection trigger the release of proinflammatory cytokines and chemokines such as TNF-α^4,19–22,37^, the attenuated pathogenicity of A(H7N9) virus infection in the CS-exposed mice might involve inflammatory signaling, especially through TNF-α-related pathways. Further studies are needed to determine the consistency and explore the mechanism of attenuated pathogenicity of A(H7N9) virus under the condition of high cytokine and chemokine expression prior to infection.

There are conflicting reports regarding the effects of CS exposure on a sublethal infection with seasonal influenza virus. Several studies have shown that mice exposed to CS had worse disease outcomes after infection with a sublethal dose of seasonal influenza virus compared to non-smoking controls^22–27^. These studies also revealed that pro-inflammatory cytokines, such as TNF-α and Il-6, were expressed at higher levels in CS-exposed, infected mice compared to air-exposed, infected mice^22,26^. These results imply that the enhanced pro-inflammatory cytokine production might cause lethal damage to the lungs of the infected mice. In contrast, another study showed that CS exposure suppresses the pro-inflammatory response after a sublethal dose of seasonal influenza virus, resulting in protective effects against influenza virus infection^29^. These conflicting reports both employed a sublethal dose of seasonal influenza virus and measured specific cytokine and chemokine responses by ELISA and/or RT-qPCR. Our sublethal challenge infection with A(H7N9) virus supported the latter report: CS exposure had a protective effect through the induction of inflammatory responses, although the virus titers in the nasal turbinates of the CS-exposed mice were higher than those of the air-exposed mice. Our microarray analysis could not explain why CS exposure switches to an antiviral or proviral effect or why virus titers in the nasal turbinates of CS-exposed mice are higher than those of controls. Further comprehensive studies are needed to answer these questions.

CS contains numerous chemical compounds that modulate gene expression in mammalian cells^39^. Among them, nicotine, a major component of CS, can suppress the immune response, including the antigen-specific immune response of T cells and the innate immune response of alveolar macrophages in vivo and ex vivo^40–42^. Han et al. report that CS exposure of mice suppressed the inflammatory response against seasonal influenza virus infection in a nicotine-dependent manner and improved body weight loss and mortality^29^. Therefore, another possibility for the attenuated pathogenicity of A(H7N9) virus in the CS-exposed mice might be the regulatory effect of nicotine on inflammatory signaling, especially the TNF-α related pathways.

Unlike seasonal H1 and H3 viruses, the impact of CS on A(H7N9) virus infection in humans was unclear because of limited epidemiological data. To rectify this, we utilized the CS-exposure mouse model and an A(H7N9) virus that lacks a furin-cleavage site in its HA protein, not a highly pathogenic A(H7N9) virus. The former mainly replicates in the respiratory tract, whereas the latter replicates in multiple organs of mice. We selected this low pathogenic A(H7N9) virus to compare the effects of CS on A(H7N9) virus to the findings of previous reports on the effects of CS against seasonal influenza viruses, which mainly replicate in the respiratory tract. Here, we found that CS exposure reduced the pathogenicity of A(H7N9) virus infection in mice. However, it is important to note that if we had used a highly pathogenic A(H7N9) virus with the furin-cleavage site, our findings may have been different.

We observed that the lungs of mice were histologically altered upon CS exposure; inflammatory cells, mainly macrophages, infiltrated the alveolar spaces. In addition, lymphoid follicles were found in the lungs of the CS-exposed mice. CS exposure induces increased permeability of epithelial cells, mucus overproduction, impaired mucociliary clearance, increased release of proinflammatory cytokines and chemokines, enhanced recruitment of macrophages and neutrophils, and disturbed lymphocyte balance towards Th2^28,43^. These infiltrating cells might secrete proteases such as matrix metalloproteinases and inflammatory cytokines and chemokines^44^, resulting in alteration of the cellular status of the lung epithelial cells. However, the mechanism of lymphoid follicle formation induced by CS exposure and the functions of lymphoid follicles remain unclear. Further analyses are required to reveal the formation process and functions of lymphoid follicles.

In summary, here, we showed that CS exposure had a protective effect against A(H7N9) virus infection through upregulated inflammatory responses prior to infection. Despite such beneficial effects, the detrimental health effects of habitual smoking in humans are proven. Therefore, we do not recommend habitual smoking at all.

## Materials

### Viruses and cells

A/Anhui/1/2013 (H7N9) ^4^virus was propagated in embryonated chicken eggs. All experiments with A(H7N9) virus were performed in enhanced biosafety level 3 (BSL3) containment laboratories at the University of Tokyo (Tokyo, Japan), which are approved for such use by the Ministry of Agriculture, Forestry, and Fisheries of Japan.

### Exposure of mice to cigarette smoke

**Fig. 6:**
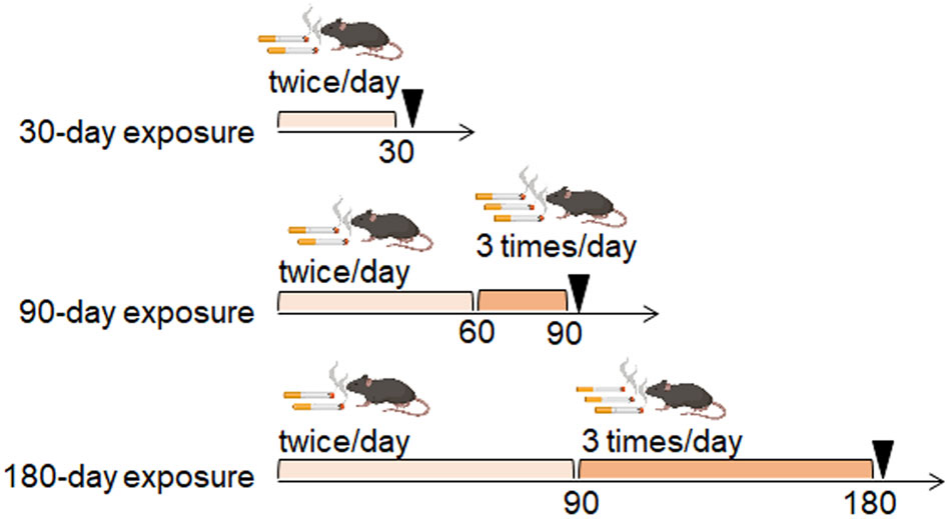
Schedule of CS exposure. For the 30-day exposure to CS group, mice were exposed to cigarette smoke twice a day. For the 90-day exposure to CS group, mice were exposed to cigarette smoke twice a day for 60 days and then three times a day for the last 30 days. For the 180-day exposure to CS group, mice were exposed to cigarette smoke twice a day for the first 90 days and then three times a day for the latter half of the experiment. After CS exposure, mice were infected with A(H7N9) virus at the timepoints indicated by the black tringles.

Seven-week-old female C57BL/6j mice were purchased from Charles River Japan Inc. (Japan). Mouse cigarette smoke exposure experiments were conducted at the CMIC Bioresearch Center (Yamanashi, Japan), an institution accredited by the Association for Assessment and Accreditation of Laboratory Animal Care International (identification number: 001182). Mice were exposed to cigarette smoke (Peace®, 28 mg of tar, 2.3 mg of nicotine/cigarette, Japan Tobacco Inc., Tokyo, Japan) or to air (as a control) for 20‒30 min per exposure time by using a mainstream smoke-generating apparatus (INH06-CIG01A, M.I.P.S. Inc., Osaka, Japan) twice or three times a day for 30–180 days (Fig. 6). Smoking frequency was set to 3 exposures per day after 60 or 90 days to enhance the effects of CS exposure.

### Infection of mice with A(H7N9) virus

Mice (*n* = 4 per group) exposed to CS were intranasally inoculated with 10^2^ or 10^3^ PFU of A(H7N9) virus in 50 μl of PBS under sevoflurane anesthesia, and body weights and survival were monitored daily for 14 days post-inoculation (dpi). Lungs and nasal turbinates were harvested from virus-infected mice (*n* = 3 per group) for virus titration, micro array analysis, and histological experiments at 2 and 5 dpi. All animal experiments were performed in accordance with the regulations of the University of Tokyo Committee for Animal Care and Use and were approved by the Animal Experiment Committee of the Institute of Medical Science of the University of Tokyo (PA13-28).

### Virus titration

The virus titers in the lungs and nasal turbinates collected at 2 and 5 dpi were determined by performing plaque assays on MDCK cells.

### Pathological examination

Excised mouse lung tissues were fixed with 4% paraformaldehyde in phosphate buffer solution. The tissues were then processed for paraffin embedding and cut into 3-μm-thick sections. The sections of each tissue sample were stained using a standard hematoxylin and eosin (H&E) procedure. Each serial section was processed for immunohistological staining with a rat anti-mouse CD45R/B220 (BD Pharminben, Cat 553084), a rat anti-mouse Mac-3 (BD Pharmingen, Cat 550292), and a rabbit polyclonal antibody for type A influenza virus nucleoprotein (NP) antigen (prepared in our laboratory). Specific antigen–antibody reactions were visualized by using 3,3’-diaminobenzidine tetrahydrochloride (DAB) and the DAKO LSAB2 system (DAKO Cytomation) as described previously^4^.

### Microarray analysis

Total RNA was extracted from lung samples by using the RNeasy Mini Kit (Qiagen), according to the manufacturer’s protocol. RNA sample was labeled with Cy3 dye with the Quick Amp labeling kit (Agilent Technologies), and hybridized to the Mouse Gene Expression Microarray (Agilent Microarray Design Identification Number 028005; Agilent Technologies), as previously described^38^. Individual microarrays were performed for each lung sample that was collected from naïve and infected animals exposed to CS or air. Statistical analysis was performed using the LIMMA package. The log2 of the intensity of each probe was background corrected and normalized between arrays (using the quantile method). Duplicate probes were averaged. Hierarchical clustering was used to review biological replicate quality. For each exposure period (30, 90, and 180 days) and day post-infection (2 and 5 dpi), a two-way ANOVA was performed with CS exposure (CS-exposed vs air-exposed) and infection status (infected vs mock) as fixed variables. Supplementary Fig. S1 shows the number of genes differentially expressed for the main effects (infected vs uninfected for exposed or unexposed animals) and the interaction effects of these two fixed variables when using the moderated t statistic for each contrast and considering a false discovery rate (FDR) < 0.05 for differentially expressed genes. To focus on genes differentially expressed based on the interaction of smoke-exposure and infection across the six groups (30-, 90-, and 180-day exposure and then sampled at 2 or 5 dpi), TopTable was used to extract the genes that were differentially expressed, which was defined as having an FDR < 0.01 when considering the F-statistic. The expression of these genes is visualized in Fig. 5. Primary gene expression data are available in Gene Expression Omnibus (series number GSE261627) in accordance with proposed Minimum Information About a Microarray Experiment (MIAME) guidelines. Mouse gene annotations for the array were provided by Agilent via Agilent’s eArray.

### Gene functional enrichment analysis

Functional enrichment analysis was performed with ToppCluster and Ingenuity’s Pathway Analysis (IPA). For ToppCluster, an enrichment score >2 (corresponding to an FDR < 0.01) was the minimum required for enrichment. A *p* value < 0.01 was required for all analysis performed with IPA.

### Statistical analysis

We used R (www.r-project.org) and lme4^45^ to perform a linear mixed effects analysis of the body weight data (normalized to the initial weight of each individual animal). As fixed effects, we used the air and CS groups, and the time of the measurement (with an interaction term between those fixed effects). As random effects we had intercepts for the individual animals. We used the lsmeans^46^ package to compare the groups at different timepoints, for each model separately, and the *p*-values were adjusted using Holm’s method. For the comparisons of virus titers of different organs at different timepoints, we used a two-way ANOVA, followed by pairwise comparisons of the groups at each timepoint. The titers measured in the lungs and in the nasal turbinates were analyzed separately. The *p*-values were adjusted using Holm’s method. For the analysis of the survival data, we used the Log-rank test, comparing the CS group to the control group. We used the OASIS 2^47^ software for this analysis.

## Supplementary information

Suppleental materials

Table S1

## Data Availability

The datasets analyzed during the current study are available in Gene Expression Omnibus (series number GSE261627). Other data are available from the corresponding author on reasonable request.

## Material availability

All reagents described in this paper are available through Material Transfer Agreements.

## Code availability

No custom code was used during the data acquisition or analysis. Code applying standard R statistical packages is available on request.
