# Supplementary material for: Attenuation of A(H7N9) influenza virus infection in mice exposed to cigarette smoke
Source: Npj Viruses. 2024 Mar 25;2:16. doi: 10.1038/s44298-024-00026-4 (PMC11721123; doi:10.1038/s44298-024-00026-4)
Supplement: Supplementary file 1 — Suppleental materials [file 44298_2024_26_MOESM1_ESM.docx]

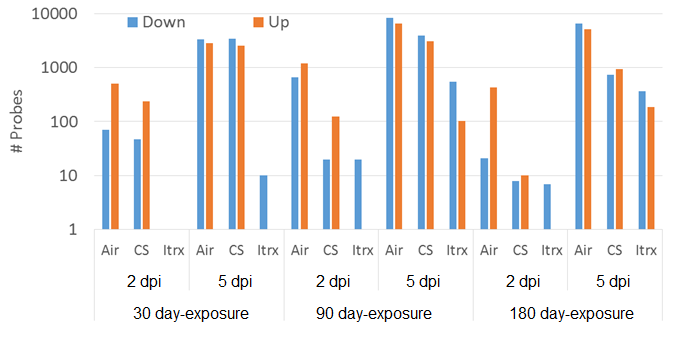


**Figure S1. Differentially expressed probes.** For each exposure period and day post-infection sampled, the main effects (e.g., infected vs mock-infected animals that were exposed to smoke for 30 days) and interaction effects (e.g., how smoking and infection impacted gene expression) were analyzed by using a two-way ANOVA. Shown are the number of differentially expressed probes when comparing infected to mock-infected for animals exposed to air or exposed to CS. Each exposure period and day sampled post-infection was analyzed separately. For each exposure period, we show the number of probes that were differentially expressed when considering interactions (Itrx) between smoking and infection status.


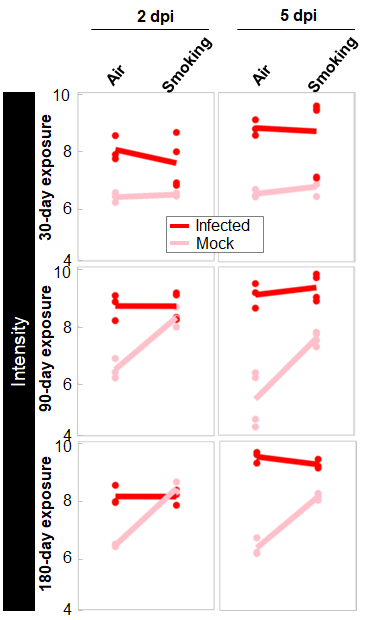


**Figure S2. Comparison of TNF-α expression between infection status and exposure history.** Data on probes that detected TNF-α expression were extracted from the microarray analysis and plotted by smoking period and infection history.


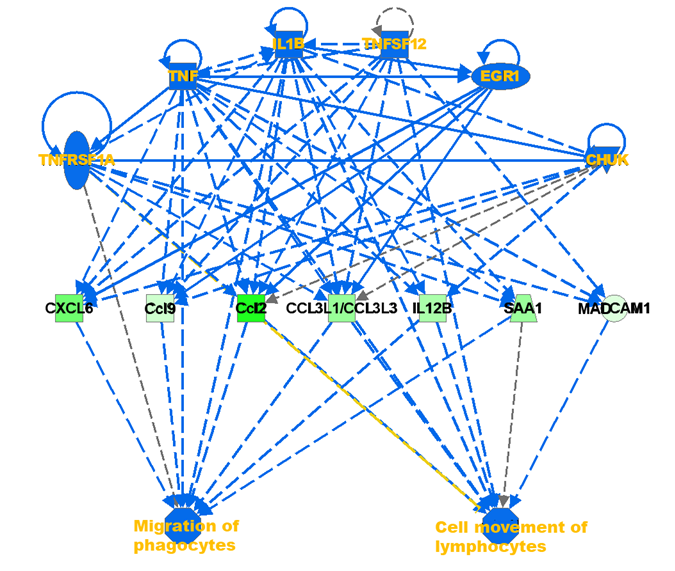


**Figure S3. Gene expression regulator analysis.** Genes that are differentially expressed when considering infection status and smoke exposure (i.e., interaction terms) were analyzed with IPA upstream regulator analysis. The analysis identified a subnetwork of interactions that may define how CS impacts infection. Each triangle, square, or circle is a protein; the hexagons represent immune cell activity.
